# Next-generation vanadium redox flow batteries: harnessing ionic liquids for enhanced performance

**DOI:** 10.1039/d5ra02901e

**Published:** 2025-07-17

**Authors:** Kalyan Sundar Krishna Chivukula, Yansong Zhao

**Affiliations:** a Department of Safety, Chemistry and Biomedical Laboratory Sciences, Western Norway University of Applied Sciences (HVL) 5063 Bergen Norway yansong.zhao@hvl.no yansong.zhao2004@gmail.com

## Abstract

Vanadium redox flow batteries (VRFBs) have emerged as a promising contenders in the field of electrochemical energy storage primarily due to their excellent energy storage capacity, scalability, and power density. However, the development of VRFBs is hindered by its limitation to dissolve diverse vanadium salts in the aqueous solution without significantly impacting the viscosity and thereby, the operational efficiency. To address this challenge, a novel aqueous ionic-liquid based electrolyte comprising 1-butyl-3-methylimidazolium chloride (BmimCl) and vanadium chloride (VCl_3_) was synthesized to enhance the solubility of the vanadium salt and aid in improving the efficiency. The synthesized novel electrolyte combination showcased a maximum theoretical energy density of approximately 44.24 Wh L^−1^, a dynamic viscosity of 36.62 mPa s along with a stable potential window of approximately 1.8 V, and an ionic conductivity of 0.201 S cm^−1^ at room temperature. Furthermore, the aqueous ionic-liquid based VRFB demonstrated an appreciable coulombic efficiency and capacity retention of greater than 85% at a discharge current of 5 mA. The maximum achievable concentration utilizing deionized water was obtained to be 2 M, which can be significantly enhanced by utilizing various component combinations of organic solvents, and ionic liquids to unlock the full potential of VRFBs. This novel electrolyte composition provides a promising pathway for improving the energy density and operational efficiency of VRFBs, paving the way for advanced energy storage solutions.

## Abstract 2

A novel acid-free VRFB electrolyte utilizing BmimCl + VCl_3_ achieves 1.8 V stability, CE > 85%, and 7.26 Wh L^−1^ energy density. Demonstrates green chemistry, enhanced vanadium solubility, and stable charge–discharge performance.

## Graphical abstract

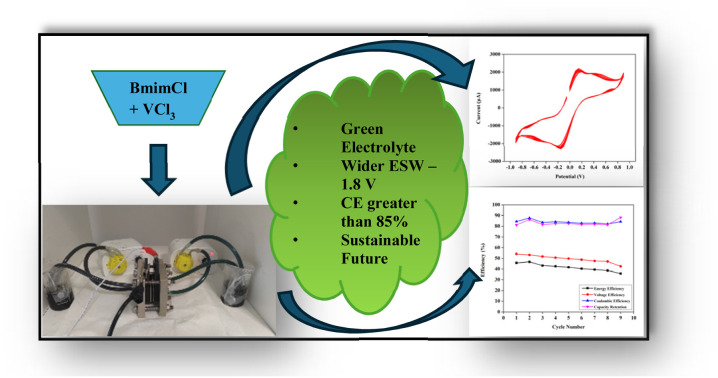

## Introduction

Energy storage technologies are pivotal in addressing the growing demand for reliable and efficient energy systems. Amongst the various electrochemical energy storage devices, batteries have emerged as key players, primarily owing to their optimal energy and power densities, as well as their capacity to store the energy efficiently, as compared to capacitors, super-capacitors, fuel cells *etc.*^1,2^ Prominent battery chemistries like lithium-ion (Li-ion), sodium-ion (Na-ion), zinc-ion, lead-acid, and the corresponding solid-state batteries have carved significant niches in this energy storage landscape.^3–7^ However, these battery systems are fundamentally limited from expanding their horizons any further, by the available active surface area of the electrode materials, the concentration of dissolved salts in the electrolytes, safety, dendrite growth, lower operational temperature window, and their conductivity across the electrolyte, constraining their overall energy storage charge and discharge capacity.

In contrast to these traditional ion-based batteries, redox flow batteries (RFBs) present an innovative solution to overcome these limitations.^8–10^ A key advantage of RFBs^11^ is that their energy storage capacity can be scaled independently *i.e.*, by increasing the volume of the liquid electrolytes which are commonly known as catholyte and anolyte. Amongst the other features, this unique ability positions RFBs as a versatile and scalable alternative to traditional battery technologies in the grid-scale energy storage.

**Fig. 1:**
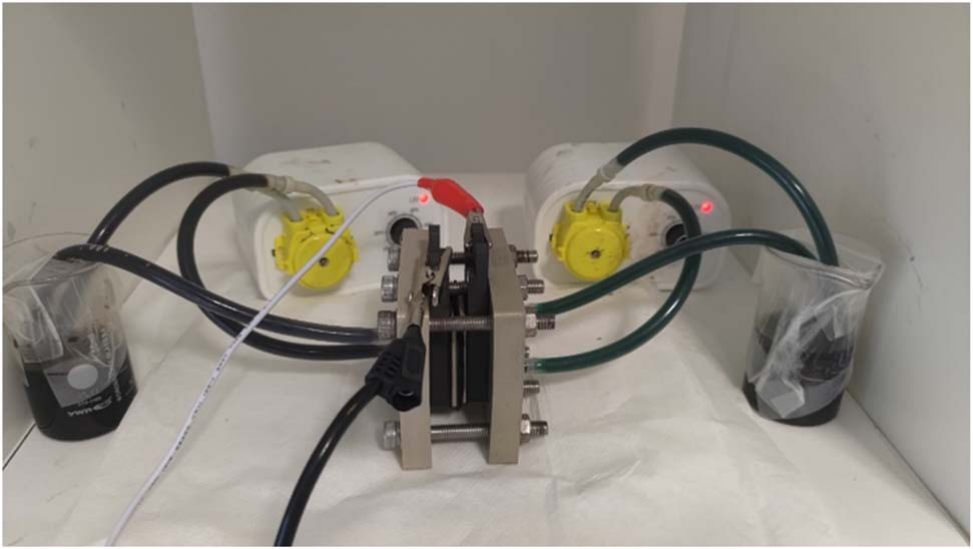
The general assembly of a redox flow battery comprising of two pumps, two beakers consisting of catholyte and anolyte (indicated by dark brown (+5), and green (+2) respectively), and a cell stack consisting of porous electrodes, membrane, and current collector plates.

At the core of RFB operation is the separation of energy storage and power generation functions, which are independent of each other. The liquid electrolytes *i.e.*, the anolytes and catholytes, are stored externally in a tanker and pumped through a cell stack containing current collectors, porous electrodes, and an ion-exchange membrane. The membrane facilitates ion transport between the anolyte and catholyte, ensuring efficient electrochemical redox reactions, and a continuous energy storage and release as shown in Fig. 1.

Among the various types of RFBs, vanadium redox flow battery (VRFB) stands out for its ability to eliminate cross-contamination between electrolytes, a common issue in other flow battery chemistries which induces self-discharge of the device. By utilizing vanadium as salt in both the anolyte and catholyte, VRFBs significantly enhance their energy storage capacity and operational stability, making them a leading contender for large-scale energy storage solutions.

In VRFBs, energy storage is achieved through the use of vanadium ions in different oxidation states ranging from +2 to +5. The positive electrolyte (catholyte) contains vanadium in the +4 (VO^2+^) and +5 (VO_2_^+^) oxidation states, while the negative electrolyte (anolyte) contains vanadium in the +2 (V^2+^) and +3 (V^3+^) states as illustrated in the Fig. 1. This all-vanadium system prevents cross-contamination, a common issue in other redox flow battery chemistries, such as iron–chromium (Fe–Cr) and bromine–polysulfide (Br–polysulfide) systems.

In a typical VRFB, vanadyl sulfate (VOSO_4_) is dissolved in sulfuric acid (H_2_SO_4_) and water to form the electrolyte. During discharge, vanadium ions at the electrodes undergo electrochemical reactions, where the carbon felt or graphite electrodes facilitate electron transfer to the external circuit, and protons move across a cation-exchange membrane (*e.g.*, Nafion) to balance charge in the system. The key half-cell reactions and their respective standard potentials (*versus* the standard hydrogen electrode, SHE) are as follows:

Anode reaction (discharge):V^2+^ ↔ V^3+^ + e^−^, *E*^0^ = −0.26 V *vs.* SHE

Cathode reaction (discharge):VO^2+^ + H_2_O ↔ VO_2_^+^ + 2H^+^ + e^−^, *E*^0^ = 1.00 V *vs.* SHE

The overall open circuit potential (OCP) of the VRFB is determined by the potential difference between the cathode and anode reactions, yielding the following,VO^2+^ + H_2_O + V^3+^ ↔ VO_2_^+^ + V^2+^ + 2H^+^, *E*^OCP^ = 1.26 V

The key to enhancing the energy storage capacity in a VRFB is increasing the concentration of dissolved vanadium salt in the electrolyte with the help of a variety of solvents ranging from aqueous, non-aqueous, and ionic liquids *etc.* In this regard, ionic liquids (ILs) have emerged as a promising class of solvents that can significantly improve electrolyte performance in VRFBs by significantly enhancing the operating temperature ranges, electrochemical stability window, efficiency, and the concentration of vanadium salt in the electrolyte.

However, limited studies have explored the use of ionic liquids in VRFBs despite of their unique chemical and physical properties.^12–16^ As mentioned earlier, traditional VRFBs often rely on aqueous sulfuric acid-based electrolytes, but these are limited by the low to moderate solubility of vanadium ions and the imminent risk of thermal precipitation of vanadium pentoxide (V_2_O_5_) at elevated temperatures, which restricts their operating window to 10–40 °C. Overcoming this, ionic liquids offer an attractive alternative primarily due to their ability to operate over a wider temperature range, their chemical stability, low volatility, and tuneable physical properties such as viscosity and conductivity.

In a study performed by Nikiforidis *et al.*^15^ a protic ionic liquid (PIL) namely PyrrH^+^CH_3_SO_3_^−^ was formulated and synthesized, which was introduced as a solvent for vanadium-based electrolytes in VRFBs, to access its impact on the electrochemical performance of the battery. By employing PILs, researchers were able to dissolve up to 6 mol L^−1^ of vanadyl sulfate (VOSO_4_) – a 2.5 times increase in the concentration of vanadium electrolyte when compared to the maximum achievable concentration with sulfuric acid with a significant drawback of higher viscosity, and lower conductivity. The PIL-based VRFBs demonstrated thermal stability across a wide range of operating temperatures (−20 °C to 80 °C), maintaining chemical stability over several weeks. Further, electrochemical tests revealed quasi-reversible redox reactions at both the anolyte and catholyte, comparable to traditional sulfuric acid electrolytes, while achieving an open circuit potential (OCP) of 1.39 V. Moreover, To investigate the influence of protic ionic liquid under the cyclic charge–discharge conditions, a redox flow battery comprising 0.5, 2.5 and 4 mol L^−1^ vanadium(iv) was dissolved in 1.5 mol L^−1^ PIL, which is then compared to a 1.5 mol L^−1^ H_2_SO_4_ electrolyte. The VRFB's electrolyte incorporated with PIL showcased an excellent cyclic stability (150+ cycles) along with energy and coulombic efficiencies of 65% and 93% respectively with a nominal capacity of 1250 mAh at a current density of 60 mA cm^−2^. This advancement underscores the potential of PILs to dramatically enhance the performance of VRFBs.

Further exploration into non-aqueous redox flow batteries (NARFBs) utilizing ionic liquids has also demonstrated promising results. One such study formed by Bahadori *et al.*^16^ evaluated the performance of non-aqueous vanadium redox flow systems based on vanadium acetylacetonate (V(acac)_3_) in 1-butyl-3-methylimidazolium bis(trifluoromethylsulfonyl)imide ([C_4_mim][NTf_2_]) and 1-butyl-1-methylpyrrolidinium bis(trifluoromethanesulfonyl)imide ([C_4_mpyr][NTf_2_]). These IL-based systems yielded a cell potential of 2.2 V, significantly higher than the currently utilized aqueous systems, with coulombic efficiencies ranging from 88 to 92% over 50 cycles. Despite the higher viscosity of the ILs, which can slow mass transport, the systems showed quasi-reversible redox behaviour similar to the PILs utilized in the previous study, for the vanadium redox couples. This study highlighted the ability of ILs to provide a broader electrochemical window, allowing for greater energy density and wider operational flexibility compared to traditional aqueous electrolytes.

Overall, these studies establish ionic liquids as a highly versatile and effective alternative to conventional aqueous electrolytes in VRFBs. By leveraging the tuneable properties of ILs, VRFBs can achieve higher vanadium solubility, better thermal stability, and enhanced electrochemical stability windows, making them ideal candidates for next-generation energy storage technologies.

Moreover, ionic liquids have found widespread use in a variety of energy storage devices, including fuel cells, lithium-ion batteries, and supercapacitors, due to their ability to enhance performance. One ionic liquid that stands out is 1-butyl-3-methylimidazolium chloride (BmimCl). In lithium-ion batteries, BmimCl has been incorporated into hybrid gel polymer electrolytes (HGPEs) consisting of PMMA–PLA doped with LiTFSI, leading to significant improvements in ionic conductivity and electrochemical stability.^17^ By interacting with the polymer matrix, BmimCl facilitates enhanced ion transport across the electrolyte region, resulting in higher charge carrier mobility and improved battery performance. The electrolyte consisting of 80% PMMA:20% PLA:20 wt% LiTFSI:15 wt% BmimCl showcased an extremely high ionic conductivity *viz.* 1.63 × 10^−3^ S cm^−1^ at room temperature. Furthermore, the electrochemical stability window of the thus-formed electrolyte was evaluated at room temperature, and it was 3.4 V *vs.* Li/Li^+^.

In the case of supercapacitors, BmimCl-based ionogels have shown excellent mechanical strength, ion conductivity, and the ability to perform at high temperatures, making them ideal for use in all-solid-state super capacitors devices.^18^ A notable combination mentioned in the study was of BmimCl with chitosan and hydroxyethyl methacrylate (HEMA), which forms an ionogel that not only recovers its mechanical strength after compression but also demonstrates superior electrochemical behaviour at elevated temperatures.

BmimCl has also proven to be a valuable component in the electrolytes in fuel-cells,^19^ particularly when used to enhance the performance of proton-conducting polymer electrolytes. Studies have shown that doping poly(vinyl alcohol) (PVA)-based membranes with BmimCl improves both the ionic conductivity (5.74 mS cm^−1^) and thermal stability (250 °C) of the fuel cells, resulting in higher efficiency and a maximum power density of 18 mW cm^2^ at room temperature. Finally, BmimCl has been successfully applied in rechargeable iron-ion batteries,^20^ where its high stability, non-volatility, and superior ionic conductivity make it a safer and more efficient alternative to traditional organic electrolytes. This makes BmimCl a versatile candidate for improving the performance of various next-generation energy storage technologies.

In this study, 1-Butyl-3-Methylimidazolium Chloride (BmimCl) is utilized in combination with Vanadium Chloride (VCl_3_), and de-ionized (DI) water, to induce a common ion in comparison with the ionic liquid, to develop an aqueous ionic liquid-based VRFB. This novel electrolyte formulation demonstrated superior cyclic charge–discharge performance at room temperature along with an optimum viscosity of the liquid electrolyte, and a broad electrochemical stability window. The replacement of BmimCl with H_2_SO_4_ offered a much safer, and an efficient alternative while maintaining high efficiency, and electrochemical performance which has not been explored as a potential electrolyte for VRFBs.

This manuscript focuses on several key aspects, including the preparation of a novel aqueous ionic liquid based electrolyte for VRFBs, and their comprehensive characterization utilizing a wide-range of techniques such as Fourier-transform infrared spectroscopy (FTIR), viscometry, density measurement, cyclic voltammetry (CV), and cyclic charge–discharge performance tests to evaluate the physical and chemical properties of the electrolyte system. These characterizations provide a detailed understanding of the enhanced electrochemical performance and stability offered by BmimCl in VRFB applications, highlighting its potential as a viable and improved alternative to traditional acid-based systems by promoting green-chemistry.

## Experimental section

The electrolyte utilized in the study was synthesized with the help of 1-butyl-3-methylimidazolium chloride (CAS R.N. 79917-90-1, BmimCl), an ionic liquid obtained from Thermo Fisher Scientific with a molecular weight of 174.67 g mol^−1^, and a purity greater than 98%. BmimCl is a widely recognized ionic liquid as mentioned in the previous section, for its role in enhancing ionic conductivity and thermal stability. Additionally, vanadium chloride (CAS R.N. 7718-98-1, VCl_3_) was utilized as the source for vanadium ions in the electrolyte formulation. Vanadium Chloride is also purchased from Thermo Fisher Scientific which has a molecular weight of 157.3 g mol^−1^, and a purity of 97%. Both the materials were utilized as received, without further purification, to maintain consistency across all tests. Apart from these materials, graphite current collector plates have been utilized in the VRFB along with carbon-felt as an electrode material. However, to maintain the consistency of the results, new electrodes have been utilized prior to evaluating the performance of the VRFB. Furthermore, a cation based ion-exchange membrane was utilized namely NAFION 212, to enhance the transport of protons from the aqueous ionic liquid based electrolyte.

### Preparation, and verification of the aqueous ionic-liquid based electrolyte

To synthesize a 1.3 mol L^−1^ BmimVCl_4_ aqueous solution, 0.04 moles of BmimCl were mixed with 0.04 moles of VCl_3_ to form 0.04 moles of BmimVCl_4_. To ensure thorough mixing of the constituents, 15 mL of ethanol was added, and the solution was stirred overnight *viz.* 8 hours, using a magnetic stirrer. Since BmimCl is sensitive to water, parafilm was utilized to tightly cover the surface of the beaker, to prevent its interaction with the atmospheric moisture content.

Further, the ethanol present in the solution was carefully removed utilizing a rotavapor system the next day. During this process, the pressure was maintained below 10 mbar at a temperature of 95 °C primarily to enhance the removal of ethanol, and the rotation speed was set at 120 rpm to facilitate a uniform solvent removal.

The removal process was monitored by measuring the weight of the solution before and after the treatment. The procedure was continued until there were no visible droplets of ethanol condensed in the collector tank. Once the ethanol was fully removed, the resulting thick and viscous mixture was verified to identify any residual presence of ethanol using Nuclear Magnetic Resonance (NMR). ^1^H Nuclear Magnetic Resonance (^1^H NMR) spectroscopy was employed to assess the chemical composition and purity of the synthesized ionic liquid. The measurements were performed on a 600 MHz spectrometer using DMSO as the solvent. The recorded spectrum displayed the following chemical shifts (*δ*, in ppm): *δ* 9.36 (s, 1H), 7.78 (d, *J* = 42.7 Hz, 2H), 4.17 (t, *J* = 6.6 Hz, 2H), 3.86 (s, 3H), 1.75 (p, *J* = 6.6 Hz, 2H), 1.24 (h, *J* = 6.9 Hz, 2H), and 0.89 (t, *J* = 7.1 Hz, 3H).

No peaks corresponding to the residual ethanol in the BmimVCl_4_ solution were observed. Ethanol typically displays a triplet around *δ* 1.2 ppm (–CH_3_) and a quartet near *δ* 3.6 ppm (–CH_2_–OH), which are clearly absent in the obtained spectrum. Additionally, a small signal at *δ* ∼3.3 ppm was attributed to water present in the deuterated DMSO (DMSO-d_6_), and not related to the sample. On conforming the purity of the electrolyte, the sample was diluted, and homogenized with deionized water to synthesize 1.3 mol L^−1^ BmimVCl_4_ aqueous solution.

A total of 30 mL of this solution was prepared and subsequently divided into two equal portions of 15 mL each. These portions were designated for use as the catholyte and anolyte in the VRFB. This careful preparation ensures the homogeneity and accuracy of the electrolyte solution, which is critical for optimal battery performance.

### Battery assembly and programme setup

The assembly of the VRFB consists of key components, as depicted in the Fig. 1. The central part of the setup as showcased in the figure, is the flow cell, which is clamped tightly utilizing stainless steel bolts and nuts to ensure that there is no leakage of the liquid electrolyte during the operation of the battery. The flow cell consists of a proton exchange membrane, NAFION – 212 membrane with an effective contact area of 4 cm^2^ (2 cm s × 2 cm s), placed between two graphite current collector plates, and porous carbon felt electrodes, which ensures the redox reactions in catholyte, and anolyte. The cell stack is connected to an external circuit using alligator clips (red, and black as depicted in the Fig. 1) to facilitate the flow of electrons between the two half-cells.

Two separate electrolyte reservoirs containing the synthesized BmimVCl_4_ electrolyte solution, designated as the catholyte and anolyte, are utilized for each half-cell of the battery, through which the redox reactions occurs while charging and discharging. Tubes connect the reservoirs holding the liquid electrolyte to the flow cell, and two positive displacement peristaltic pumps are employed to circulate the electrolytes continuously through the flow cell, ensuring that the redox-active species reach the porous carbon felt electrodes in the flow cell. The flow rate of the catholyte, and the anolyte solutions has been fixed at 20% of the overall RPM of the pump, which corresponds to a mass flow rate of 0.21 g s^−1^.

The experimental setup is carefully arranged to maintain a stable flow of electrolyte with the help of a positive displacement peristaltic pumps. The system is tested for a series of charge and discharge cycles with varying voltage, current conditions to assess the overall efficiency of VRFB.

The programme followed during the charge and discharge cycles is as outlined below,

(1) Rest period (30 seconds): at the start of the testing cycle, the system is allowed to rest for 30 seconds prior to the first charge cycle, to ensure that the open-circuit potential (OCP) is as close to zero as possible. This process aids in eliminating any residual charges in the system, and ensures an accurate efficiency measurement.

(2) Charging at constant current (25 mA): the first charging step is performed at a constant current of 25 mA until the voltage of the cell reaches 1.5 V. The voltage is restricted to 1.5 V to prevent the electrolysis of water in the aqueous ionic liquid based electrolyte, which could otherwise negatively impact battery performance by releasing hydrogen, and oxygen gases at the anode, and cathode respectively, and cause significant performance degradation over time.

(3) Charging at constant voltage (1.5 V): on reaching the potential difference of 1.5 V, the charging process now proceeds in a constant voltage regime which is significantly slower as compared to a constant current regime. At a constant voltage of 1.5 V the battery charges till the capacity reaches 100 mAh. This ensures that an adequate amount of charge is stored for the subsequent efficiency calculations and performance evaluation throughout the cycles.

(4) Rest period (10 seconds): once the charging phase is complete *i.e.*, the CC and CV regimes, the system proceeds to rests for 10 seconds, which allows the cell potential to stabilize, and the discharge process is initiated. The resting cell voltage is typically around 1.3 V and is recorded for later analysis.

(5) Discharge at constant current (1 mA): the discharge phase begins at a constant current of 1 mA until the voltage reaches to 0.5 V. This controlled discharge at low discharge currents allows for a steady release of stored energy from the battery.

(6) Discharge at constant voltage (0.5 V): once the voltage reaches 0.5 V, the discharge continues at the constant voltage of 0.5 V until the current drops below 0.1 mA. This step ensures the battery is fully discharged, allowing for accurate energy efficiency calculations.

(7) Cycle repetition: this entire charge–discharge process is repeated for approximately 10 cycles to measure the efficiency and performance variation of the VRFB with cycle number. This is essential for determining the durability and operational stability of the battery over time.

Unless otherwise specified, throughout the manuscript, the following programme has been utilized to charge and discharge the VRFB, to measure its electrochemical characteristics.

### Viscosity measurement

To achieve an optimal viscosity for the BmimVCl_4_ aqueous solution, approximately 45 wt% of deionized (DI) water was added to the highly viscous, and thick mixture. The viscosity of the resulting solution was measured using a Rolling Ball Viscometer (Lovis 2000 M/ME) with a 1.8 mm capillary and a gold ball (due to its non-reactive properties with the liquid electrolyte, unlike the steel ball). This choice ensured greater accuracy in the viscosity measurements.

The measurements were conducted over a wide range of temperatures from 25 °C to 50 °C with a 5 °C step size, and at a measurement angle of 45° to account for the solution's behaviour across typical operating conditions. The temperature-dependent viscosity data provided valuable insights into the solution's flow properties, ensuring it remains within the optimal range for effective performance in the VRFB.

### Density measurement

Prior to measuring the viscosity of the aqueous ionic liquid based electrolyte, it is essential to determine its density at various temperatures. The Anton Paar Rolling Ball Viscometer (Lovis 2000 M/ME) provides the advantage of measuring both viscosity and density simultaneously. During the viscosity measurement, the instrument also records the density of the sample across a temperature range of 25 °C to 50 °C, with measurements taken at 5 °C intervals.

### Charge–discharge of the VRFB

The charge and discharge characteristics of the VRFB are measured with the help of an instrument called Landt Battery Tester CT2001A. This instrument is programmed to follow a predefined testing protocol as mentioned in the earlier sub-section, which cycles the VRFB between charge and discharge phases at varying current, voltages and capacities to assess the efficiency, and capacity variation during its cycling process. During the charge phase, a constant current of 25 mA is applied to the cell till it reaches 1.5 V, and the corresponding potential difference of the flow battery is measured and recorded. During the discharge phase, dissimilar to a charging phase, a low current of 1 mA was provided to evaluate its plateau regime, and mid-voltage precisely. Moreover, the VRFB has been charged and discharged till various capacities apart from 100 mAh, to evaluate its efficiency variation at higher discharge currents and capacities. Later on, the data on the charge and discharge characteristics has been collected to calculate the energy efficiency, energy density, and overall capacity retention over multiple cycles. The Landt CT2001A battery tester allows for precise control and monitoring of the battery's performance over extended cycling tests.

### Cyclic voltammetry test

The cyclic voltammetry test has been performed with the help of the Palmsens MultiTrace 4. A symmetrical T-cell has been prepared comprising of Ni foils, and a membrane immersed in the solution of 1.3 M BmimVCl_4_ solution. Later, the T-cell was subjected to undergo an oscillation with varying voltages ranging from −0.9 V to 0.9 V and 0.67 V to −0.67 V, with a range of scan rates ranging from 0.01 V s^−1^ to 0.05 V s^−1^ for numerous cycles to evaluate the cyclic stability, and the working potential window.

### Electrochemical impedance spectroscopy test

Similar to the CV measurement, the electrochemical impedance spectroscopy (EIS) test was performed using the Palmsens MultiTrace 4 system. A symmetrical T-cell configuration was prepared, consisting of Ni foils and a membrane immersed in the electrolyte solution. The T-cell was subjected to an EIS test over a frequency range from 0.01 Hz to 200 kHz with an AC potential perturbation of 5 mV amplitude, which is then fit with the equivalent circuit model.

## Results and discussion

### FTIR analysis

**Fig. 2:**
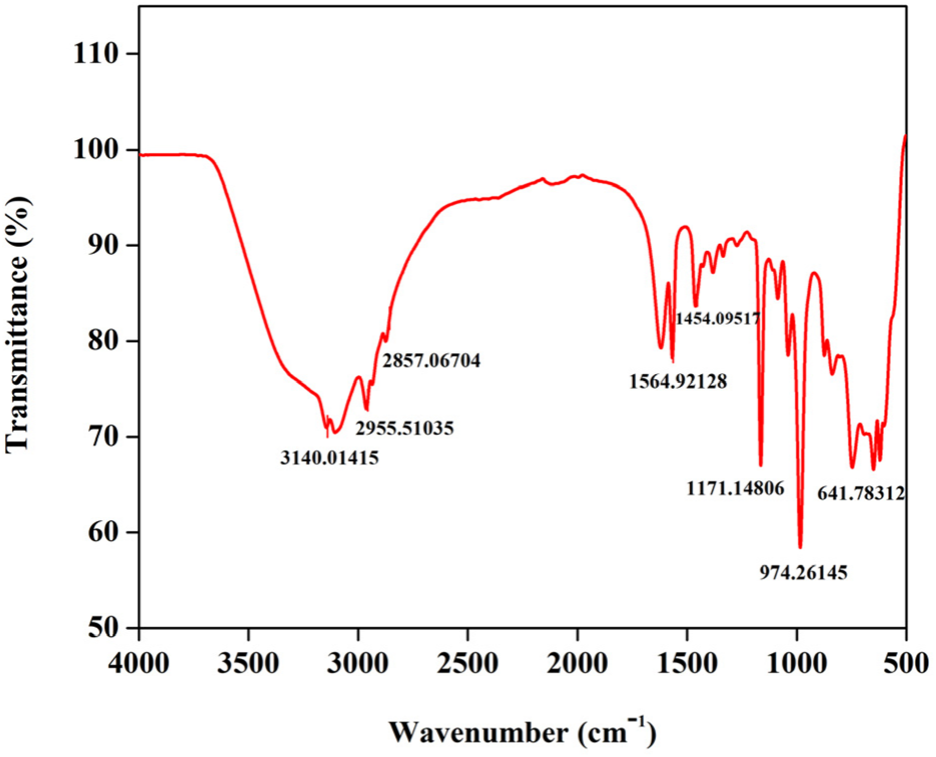
FTIR spectra of BmimVCl_4_.

Prior to evaluating the performance characteristics of the synthesized electrolyte *viz.* 1.3 M BmimVCl_4_, it is essential to identify, and to analyse the presence functional groups, and their interactions in the mixture of BmimCl and VCl_3_, the ionic-liquid based electrolyte. In order to achieve this, Fourier Transform Infrared spectroscopy (FTIR) was utilized in the study and the obtained graph is showcased in the Fig. 2. From the figure, it can be depicted that, the characteristic peaks obtained at 2955 cm^−1^, and 2857 cm^−1^, represent the aliphatic asymmetric and symmetric C–H stretching, and the peaks at and around 1564 cm^−1^ denotes the presence of imidazolium ring *viz.* C=C and C=N confirming the presence of the 1-butyl-3-methylimidazolium chloride structure, and the peaks at around 974 cm^−1^ and 641 cm^−1^ denote the presence of the vanadyl group *i.e.*, the V–O and V=O groups.^21^ Moreover, due to the moisture sensitive nature of the BmimCl ionic liquid, there appears to be a broader peak around 3100–3200 cm^−1^. This confirms the presence of BmimVCl_4_ in the synthesized electrolyte with a stable configuration between BmimCl and VCl_3_, which was later utilized to evaluate the electrochemical performance in the VRFB. However, prior to the cyclic charge–discharge testing, the CV was performed along with EIS, and physical property tests, to evaluate the extent to which it can be utilized in VRFB.

### Cyclic voltammetry test of the VRFB

**Fig. 3:**
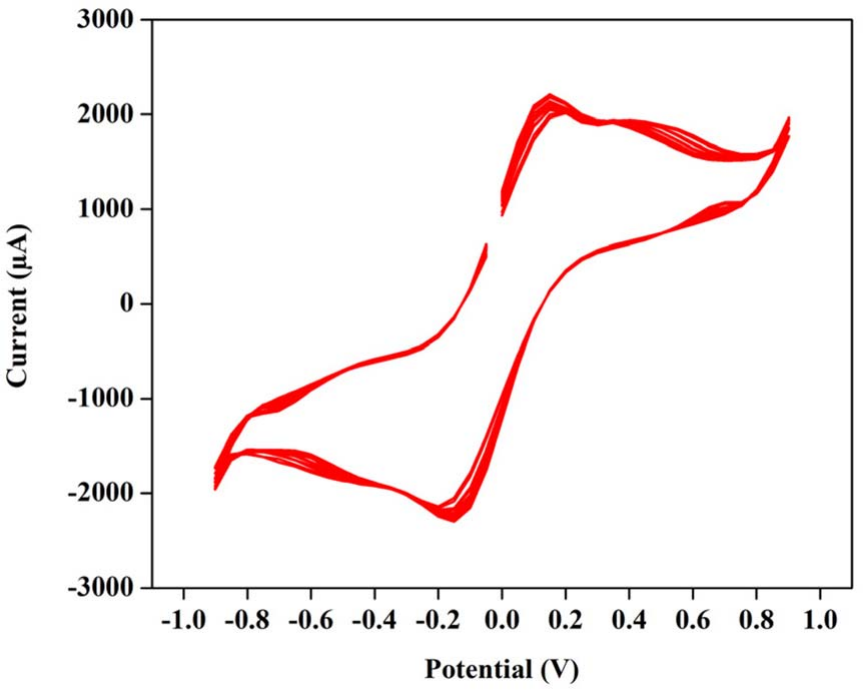
The cyclic voltammetry of 1.3 M BmimVCl_4_ at a scan rate of 0.01 V s^−1^, and voltage ranges −0.9 V to 0.9 V consisting of 20 cycles.

**Fig. 4:**
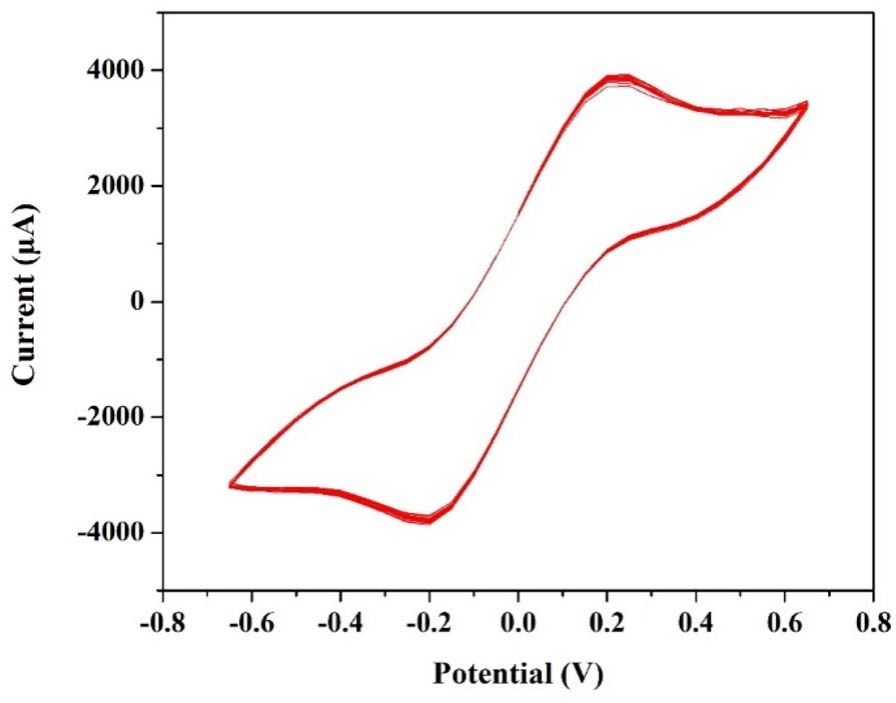
The cyclic voltammetry of 1.3 M BmimVCl_4_ at a scan rate of 0.01 V s^−1^, and voltage ranges −0.67 V to 0.67 V.

**Fig. 5:**
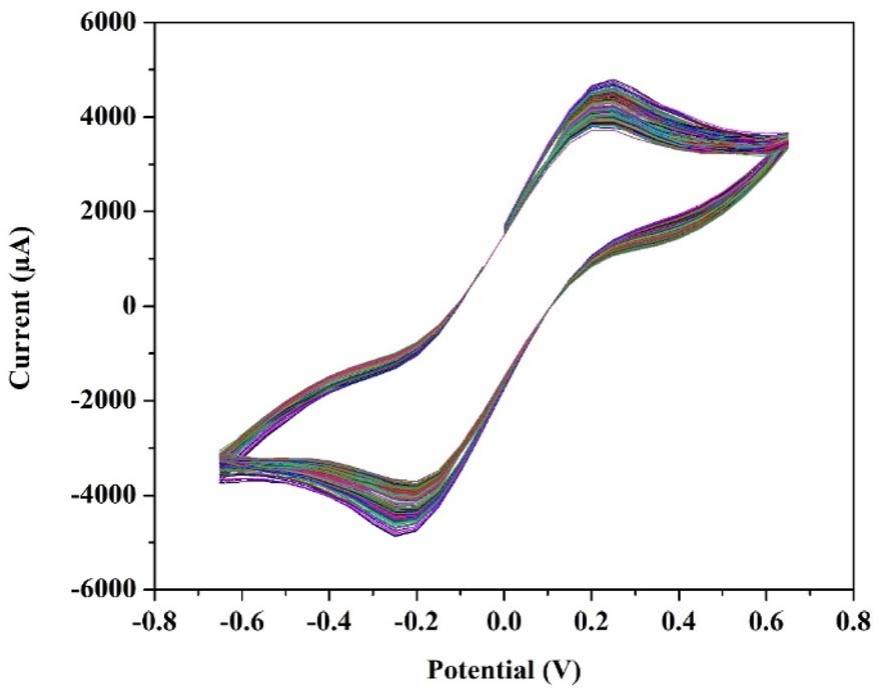
The cyclic stability of 1.3 M BmimVCl_4_ at a scan rate of 0.01 V s^−1^, and voltage ranges −0.67 V to 0.67 V.

**Fig. 6:**
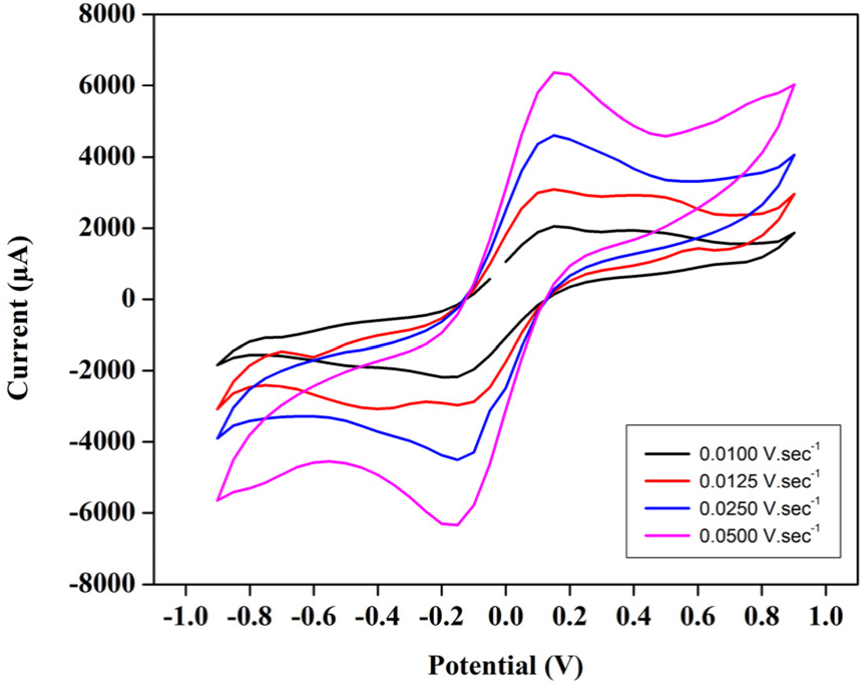
The variation of peak-current and the corresponding voltages in the CV study oscillating between −0.9 V to 0.9 V, under the influence of differential scan-rates ranging from 0.01 V s^−1^ to 0.05 V s^−1^.

The cyclic voltammetry test for a T-cell comprising of symmetric Ni foils, and a membrane immersed in the solution of 1.3 M BmimVCl_4_ was conducted for various scan rates, and potential windows as illustrated in the Fig. 3–6. Specifically, Fig. 3 and 4 depict the operational behaviour of the VRFB under varying potential windows of 0.9 V to −0.9 V and 0.67 V to −0.67 V, respectively. As depicted in the Fig. 3 and 4, there are two prominent peaks representing the oxidation and reduction of vanadium in the low voltage zones, approximately between 0.3 V to −0.3 V.

Additionally, in the Fig. 3, two more smaller oxidation and reduction peaks can be observed in the high-voltage zones. These 4 peaks suggest the reduction and oxidation of vanadium from +2 to +3, and +4 and +5. Furthermore, the theoretical open-circuit potential (OCP) of the VRFB is approximately 1.3 V (according to the thermodynamic constraint), and the experimentally obtained one is 1.27 V, which aligns closely with the voltage measured at the battery terminals when fully charged till 1.5 V. This agreement indicates the completion of oxidation and reduction reactions, confirming the reversibility of the electrochemical processes in the system.

Moreover, in order to evaluate the cyclic stability of the synthesized VRFB, a CV test has been conducted for approximately 255 cycles at a scan rate of 0.01 V s^−1^ between the voltage ranges −0.67 V to 0.67 V as illustrated in the Fig. 5. This denotes a safe working potential of approximately 1.5 V, till which the galvanostatic charge–discharge tests have been performed. Furthermore, the peaks current and voltage variation at various scan rates ranging from 0.01 to 0.05 V s^−1^ have been recorded, and illustrated in the Fig. 6.

### Electrochemical impedance spectroscopy and circuit fitting

**Fig. 7:**
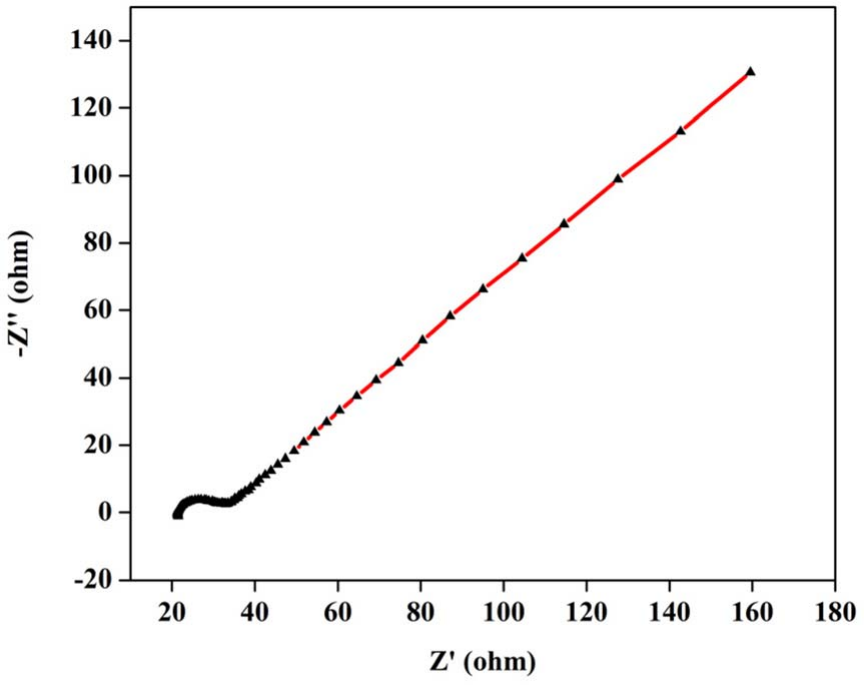
The variation of imaginary, and the real impedance with frequencies ranging from 0.01 Hz to 200 KHz.

**Fig. 8:**
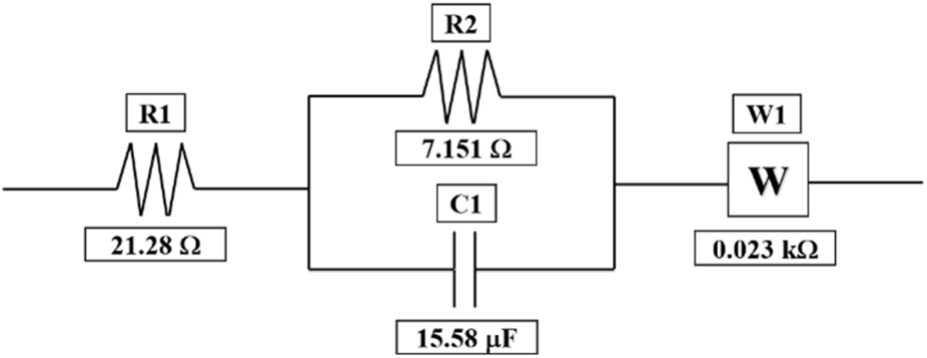
The equivalent circuit fitting along with their values for the obtained EIS data.

Furthermore, to evaluate the resistance of the electrolyte, charge-transfer kinetics, and mass-transport resistances in the cell, EIS studies have been performed. The Nyquist plot for the 1.3 M BmimVCl_4_ has been obtained from frequencies varying between 0.01 Hz to 200 kHz depicting the high, mid, and low frequency ranges to evaluate the electrolyte, and charge-transfer resistance along with the mass-transport kinetics of the electrolyte. As illustrated in the Fig. 7, a well-defined semicircle was obtained in the high frequency ranges with an *x*-axis intercept of 21.28 Ω. The resistance value of the electrolyte can be further utilized to evaluate the ionic-conductivity of the electrolyte at room temperature by utilizing the area of the electrode, length of the T-cell, and the resistance of the electrolyte. On performing the necessary calculations, the ionic-conductivity of the electrolyte at room temperature was obtained to be 0.201 S cm^−1^. Moreover, on performing the curve-fitting operations, the equivalent circuit model has been obtained which is depicted in the Fig. 8. The charge-transfer resistance between the electrode, and the electrolyte was obtained to be approximately 7.15 Ω, with a double-layer capacitance of 15.58 μF from the mid-frequency ranges, indicated by a semi-circle. The relatively low value of charge-transfer resistance indicates efficient redox reactions between the electrode, and the electrolyte region. Furthermore, the diffusion region *i.e.*, mass-transport dominated region, observed in the low-frequency zone, was fitted using a Warburg element (45°), with the associated resistance determined to be 0.023 kΩ. The fitted values, and their errors are depicted in the Table 1, wherein, the chi-squared value was obtained to 0.0006, denoting a good fit between the experimental data, and the simulated values.

**Table 1:** The fitted values of the various elements utilized in the circuit-modelling of the obtained EIS data between the frequency ranges 0.01 Hz to 200 KHz and their errors along with the chi-squared value

Element	Fitted value	Unit	Error (%)
R1	21.28	Ω	0.596
R2	7.151	Ω	2.560
C1	15.58	μF	7.112
W1	0.023	kΩ	0.916
Chi-squared	0.0006	Iterations	25

### Linear sweep voltammetry to measure the ESW

**Fig. 9:**
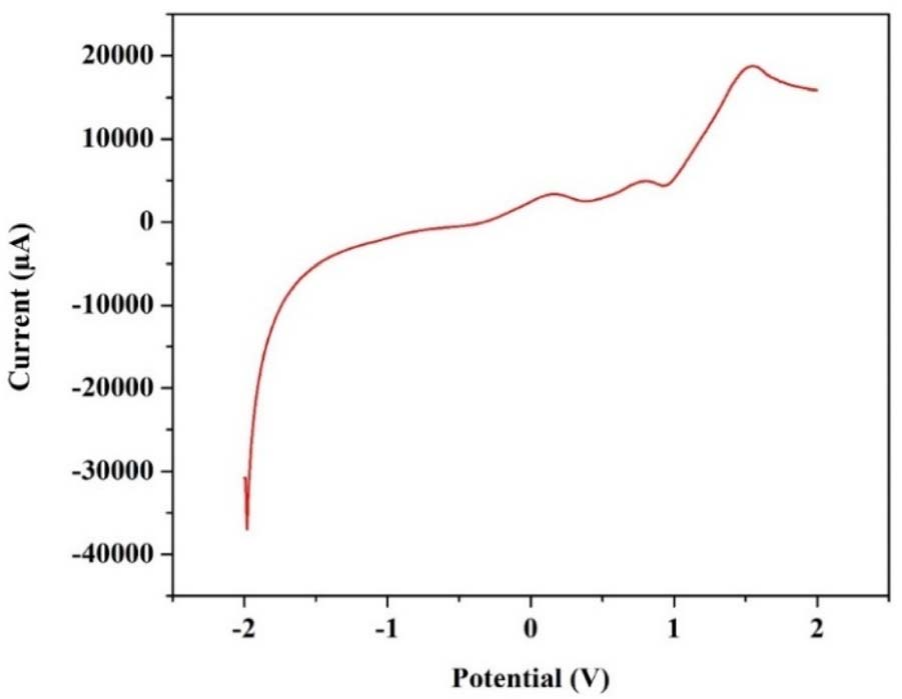
The LSV graph obtained for the electrolyte to measure its stability window, performed at a scan rate of 0.001 V s^−1^.

Furthermore, in order to evaluate the ESW of the synthesized ionic-liquid based electrolyte, liner sweep voltammetry (LSV) has been performed at a scan rate of 0.001 V s^−1^ between the potential window of −2 V to 2 V, and the obtained graph has been depicted in the Fig. 9. In correlation with the CV data, the safe operational window of the VRFB utilizing the synthesised electrolyte can be approximate to 1.5 V due to the presence of water, which undergoes electrolysis beyond 1.5 V as showcased in the Fig. 9.

### Viscosity and density of the electrolyte

**Fig. 10:**
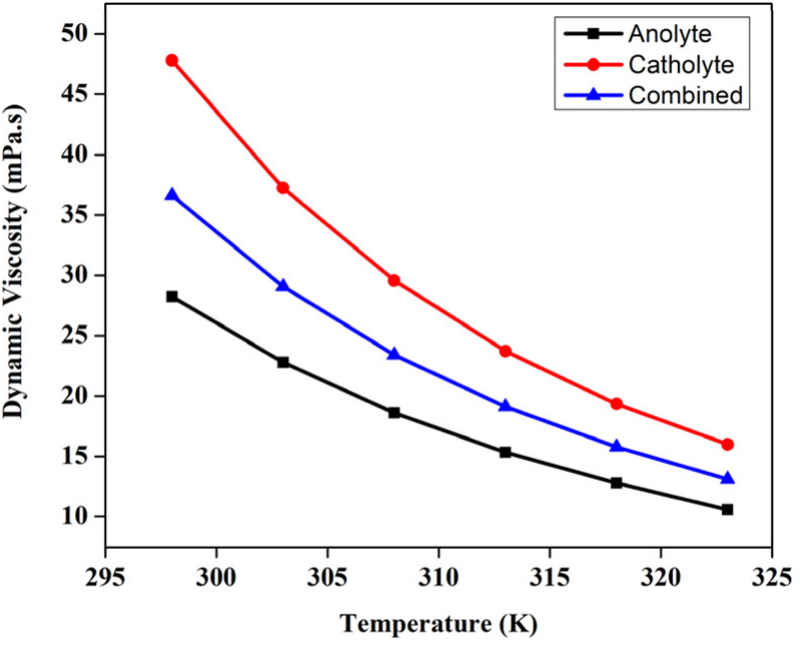
The variation in the dynamic viscosity of the anolyte, catholyte, and the mixed solution after the 20th cycle from temperatures ranging from 298 K to 323 K.

**Fig. 11:**
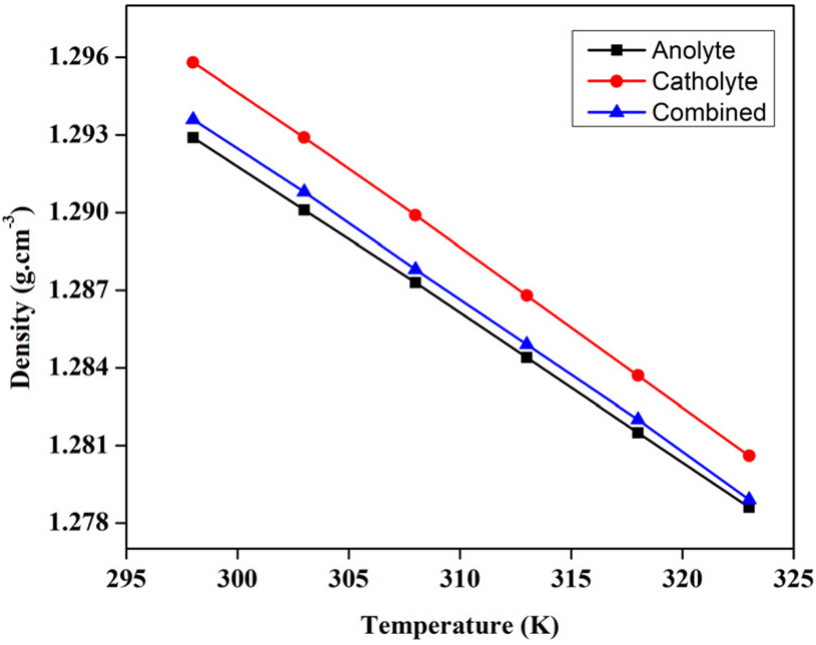
The variation in the density of the liquid electrolyte *viz.* 1.3 M BmimVCl_4_ from temperatures ranging from 298 K to 323 K.

The viscosity of the liquid electrolytes *i.e.*, the catholyte, and anolyte, play a crucial role in determining the flow characterises of the VRFB across the porous electrode region, which in turn significantly alter the efficiency of the battery. So, the viscosity of the catholyte, and anolyte, have been measured after approximately 20 cycles from various temperatures, ranging from 298 K to 323 K. Apart from the individual catholyte, and anolyte's viscosity, the dynamic viscosity of the mixture *i.e.*, the 1.3 M BmimVCl_4,_ as prepared, has been measured and depicted in the Fig. 10. The density of the same, has been reported in the Fig. 11, measured across the similar temperature ranges. The dynamic viscosity of the liquid electrolyte at the room temperature has been measured to be 28.21 mPa s, 47.81 mPa s, and 36.62 mPa s respectively for anolyte, catholyte, and the mixture, which is comparatively less as compared to the traditional ionic-liquid based electrolytes. The lower viscosity aids significantly in the convection of electrolyte which in turn enhances the efficiency of the VRFB by providing an unhindered ionic drift across the liquid electrolyte region to the porous electrodes. The decrease in the viscosity of the electrolyte with respect to the temperature ranging from 298 K to 323 K displays the conventional dynamic viscosity *vs.* temperature trend for the ionic liquids based electrolytes.

### Identification of redox mechanism, and charge–discharge characteristics of VRFB

Prior to evaluating the performance metrics offered by the 1.3 M BmimVCl_4_ solution as catholyte, and anolyte solutions, the reactions occurring between them, and the presence of various complexes along with their pH values have been studied. So, BmimVCl_4_ solutions ranging from 2.0–1.0 M have been synthesized utilizing DI water as solvent, and the corresponding pH values have been obtained subsequent to calibrating the instrument at a pH of 4, and 7, and the obtained results are illustrated in the Table 2.

**Table 2:** pH values of BmimVCl_4_ solution at various concentrations ranging from 1.0 M to 2.0 M in DI water

BmimVCl_4_ concentration (M)	Measured pH	Calculated [H^+^]/mol L^−1^
1.0	1.065	8.6 × 10^−2^
1.3	0.888	1.3 × 10^−1^
1.5	0.780	1.6 × 10^−1^
2.0	0.595	2.5 × 10^−1^

The incorporation of BmimCl to VCl_3_, results in the formation of chloro-metalate anions,^22^ which can be described as,BmimCl + VCl_3_ → Bmim^+^ [VCl_4_]^−^

The formed [VCl_4_]^−^ reacts with H_2_O in order to form the following,[VCl_4_]^−^ + H_2_O → VO^2+^ + 4Cl^−^ + 2H^+^

**Fig. 12:**
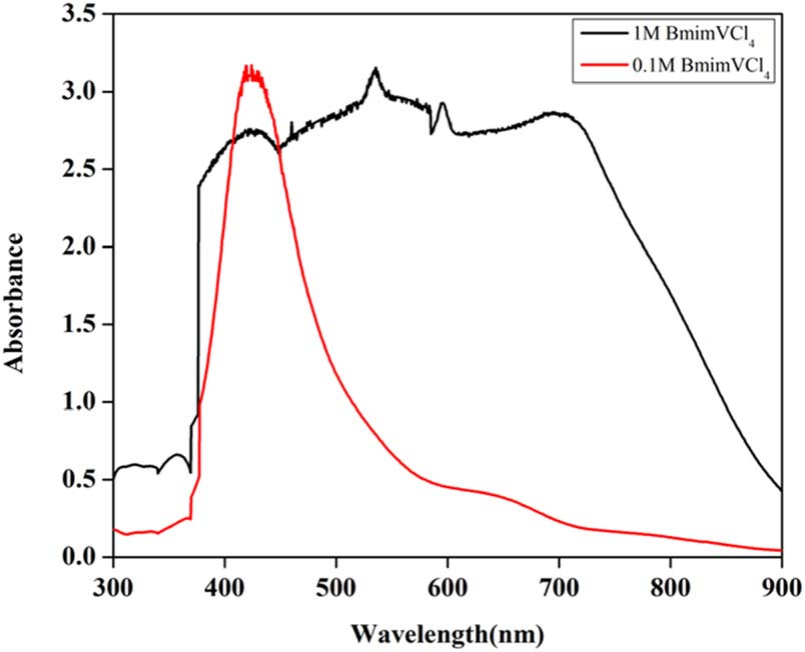
The UV-Vis data of 1 M, and 0.1 M solution of BmimVCl_4_ in the presence of DI water as solvent.

Upon the addition of DI water to reduce the viscosity of the 2.0 M BmimVCl_4_ solution, the pH has been altered, and the for the 1.3 M solution, the pH obtained was 0.888. The H^+^ ions aids in maintaining the acidic nature of the electrolyte, thereby suppressing the precipitation of V^5+^, and moreover helps in maintaining electroneutrality by transporting across the proton exchange membrane – NAFION. VCl_3_ acts as a Lewis acid (e^−^ pair acceptor), and the Cl^−^ from the BmimCl acts as a Lewis base (e^−^ pair donor), and the reaction between them leads to the formation of chloro-metalate anions as mentioned above. The formed [VCl_4_]^−^ complex is stabilised by the Bmim^+^ in the electrolyte solution. This is similar to the behaviour exhibited by AlCl_3_, FeCl_3_ in the presence of BmimCl. Further, UV-Vis studies have been conducted to assess the oxidation states exhibited by the vanadium in the solution in the form of vanadium complexes, and the obtained data is illustrated in the Fig. 12.

**Fig. 13:**
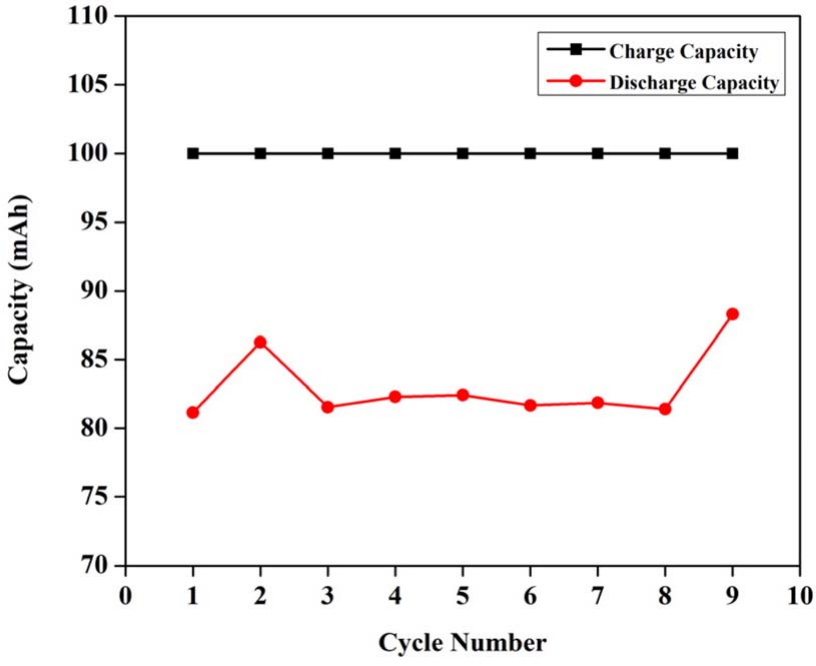
The variation of charge, and the discharge capacities of the VRFB with cycle number, wherein, the capacities are measured in mAh.

**Fig. 14:**
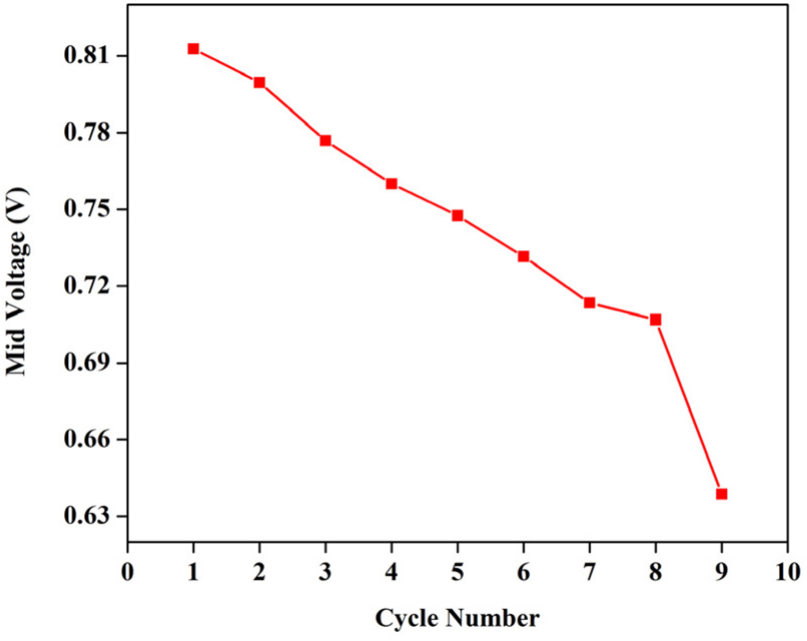
The variation of mid-voltage of the VRFB with cycle number.

**Fig. 15:**
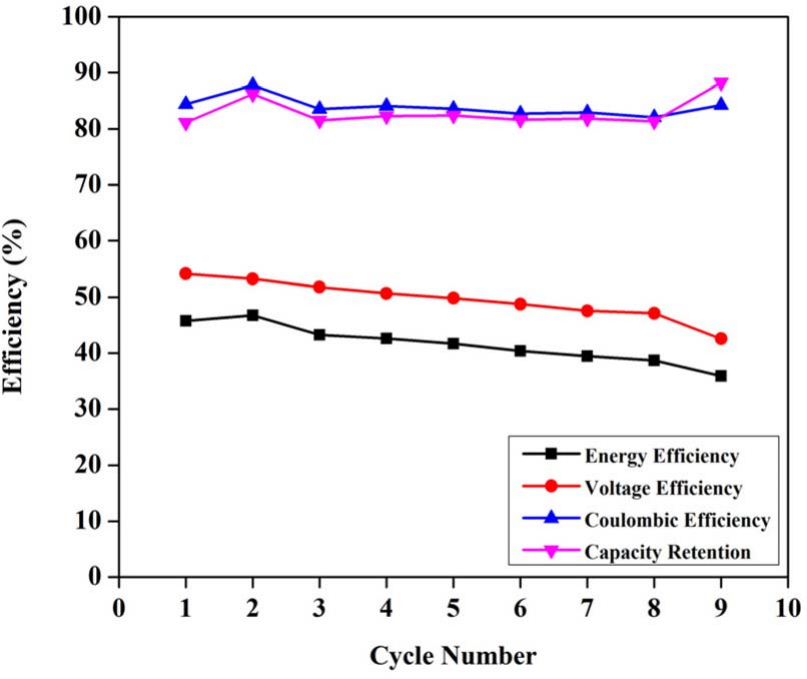
The variation of efficiencies of the VRFB with cycle number.

If the reactions proceeded in the way described above, upon the addition of DI water, V^4+^ must be formed *viz.* VO^2+^, and it has a peak around 760 nm.^23,24^ However, the 1 M solution of BmimVCl_4_ showcased a broad range of absorption ranging from 380–790 nm, indicating the co-existence of vanadium in 3 different oxidation *i.e.*, +3, +4, and +5 in the form of chloro-metalate complexes on the reaction with BmimCl. Due to the high concentration of H^+^ ions for the solutions greater than or equal to 1 M, the formation of VO^2+^ is inhibited as the reactions tends to move towards the reactants side to maintain the equilibrium according to the Le Chatelier's principle, favouring the formation of vanadium complexes rather than V^4+^, thus, leading to a broad range of absorption rather than a single peak at 760 nm. However, upon the addition of water *i.e.*, diluting the solution to 0.1 M, the concentration of H^+^ ions goes down drastically reducing the pH of the solution, and thereby favouring the production of V^4+^, which oxides to form V^5+^ in the presence of oxygen, and this is evident from the singular peak obtained at 420 nm indicating the presence of V^5+^ in the solution. To assess the impact of the synthesized electrolyte on the electrochemical performance of the redox flow battery, 1.3 M solution has been selected primarily due to its optimal viscosity, and pH values as compared to the higher concentrations. The charge–discharge characteristics of the VRFB utilizing BmimVCl_4_ as the liquid electrolyte, are illustrated in Fig. 13–15. These figures provide insights into the charge and discharge capacity, efficiency, and mid voltage variation over multiple cycles. Fig. 13 depicts the variation in charge and discharge capacities measured in mAh as a function of cycle number, wherein the charging capacity of the VRFB remains constant at 100 mAh throughout the preliminary test to maintain a consistency in the obtained results.

While the discharge capacity initially fluctuates between 81 mAh and 86 mAh during the initial cycles, after the third cycle the discharge capacity stabilizes with a mid-voltage of approximately 0.7 V, as depicted in the Fig. 14, indicating that the system has reached its stable working operational condition. As illustrated in the Fig. 15, the capacity retention of the VRFB over the different cycles alters around 83% throughout the cycles, however, the voltage efficiency (VE) lingers around 50% signifying the high resistance in the battery, resulting in significant drop of the nominal potential. The coulombic efficiency (CE) retains around 80%, and the energy efficiency (EE) remains around 45%. The following parameters were calculated as follows,
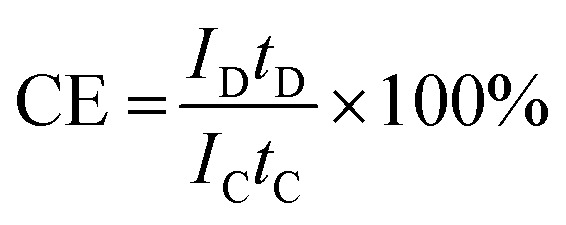

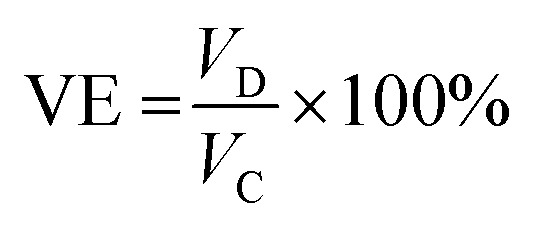
EE = CE × VEwherein, *I*_D_, *t*_D_ are the discharge currents, and time of discharge, and *I*_C_, *t*_C_ are the charging currents and time taken for the VRFB to completely charge. The terms *V*_D_ and *V*_C_ represent the discharging, and the charging potential of the VRFB.

Following the third cycle, the capacity retention stabilizes and remains consistently above 80% in subsequent cycles, confirming the reliable and stable operation of the VRFB. Further, the charging capacity has been increased to 200, 500, and 750 mAh to evaluate its influence on the discharge characteristics of the VRFB under higher discharge currents and capacities. On charging the VRFB to 200 mAh, the corresponding discharge capacity was achieved to be 182 mAh with a lower cut-off potential as 0 V, resulting in an efficiency of 91.43%, at a mid-voltage of 0.6585 V, and a discharge current of 1.5 mA. In order to obtain the maximum energy storage capacity, the battery has been further charged till 500 mAh, and 750 mAh, to evaluate its efficiency variation, and to calculate the energy density of the battery. The corresponding discharge capacities were 450 mAh, and 543.61 mAh, with efficiencies of 90% and 72.13% respectively.

As mentioned earlier, the catholyte, and the anolyte volume utilized is 15 mL, however, for the energy density calculations, 12 mL of each electrolyte has been utilized to obtain the results. The specific capacity calculated based on the highest discharge capacity was 22.6504 Ah L^−1^. This specific capacity is achieved with an efficiency greater than 70% at a discharge current of 5 mA, which is a favourable performance for VRFBs utilizing ionic liquid electrolytes for grid-scale energy storage solutions. Moreover, the energy density can be evaluated with the help of the obtained specific volumetric capacity and the nominal working potential of the VRFB as, 22.6504 Ah L^−1^ × 0.3206 V = 7.2617 Wh L^−1^, which is around approximately 17% of the maximum achievable theoretical energy density of 44.24 Wh L^−1^. The theoretical energy density has been calculated as follows,
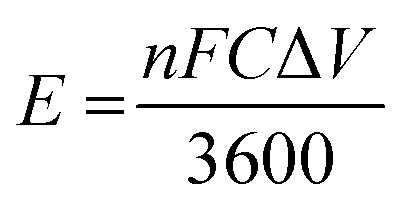
wherein, *n* denotes the number of electrons transferred during the redox reaction, *F* is the Faraday constant (96 485 C mol^−1^), *C* is the concentration of vanadium ions in the catholyte, and anolyte solutions (mol L^−1^), and Δ*V* denotes the potential of the cell (V).

The discrepancy obtained in the resulting value of energy density is primarily owed to two factors – lower value of discharge capacity, and nominal voltage during the discharge, which can be linked to the availability of V^5+^ during the discharge *i.e.*, SOC, and the mass transport resistance induced by the synthesized electrolyte. As mentioned earlier, the viscosity of the electrolyte is 36.62 mPa s at room temperature, which is significantly higher as compared to the commercialised redox flow battery electrolytes *i.e.*, VOSO_4_ in H_2_SO_4_ and DI water. The high viscosity hinders the mass transport, and thereby results in an elevated resistance for the electrolyte solution as evaluated from the EIS studies. Furthermore, as observed from the VE data, there is a significant loss *i.e.*, approximately 50%, primarily owing to the ohmic drops, and the mass transport overpotential which are in turn related to the viscosity of the electrolyte. Due to the low operational potential, the energy density of the battery is significantly reduced. Even though, subsequent to increasing the nominal potential during from 0.3 V to 1.0 V (theoretically, for the sake of evaluation), the energy density of the flow battery can reach 22 Wh L^−1^, which is almost 40% lower as compared to the theoretical energy density, and this can be attributed to the SOC of the battery *i.e.*, the theoretical assumption of 100% V^5+^ ions, must be achieved experimentally *viz.* a SOC of 100%. The future works includes reducing the viscosity of the electrolyte, and thereby suppressing the resistances in the electrolyte and enhancing the nominal discharge potential to achieve an energy density value as closer to the theoretically evaluated one.

Moreover, in comparison to a commercialised vanadium redox flow battery, the synthesized flow battery based on ionic liquid excels in the replacement of acid–base (H_2_SO_4_, HCl) systems, with a novel, green ionic liquid based electrolyte. This aligns with the green chemistry principles and address safety, environmental, and material degradation concerns that limit long-term stability in the commercial VRFBs. While ionic liquid systems offer unique benefits such as a broader electrochemical window (up to 1.8 V as demonstrated from the Fig. 3, in spite of utilizing DI water as solvent) they also introduce huge challenges on the research point of view, such as high viscosity *viz.* 36.62 mPa s, which increase the mass transport resistance and reduce the VE, and subsequently EE. Consequently, although the flow battery achieves a CE exceeding 85%, the VE remains below 60%. Furthermore, commercial VRFBs typically operate at a current density of 30–50 mA cm^−2^, while the current ionic-liquid based system is optimized for up to 1–5 mA cm^−2^. In terms of the energy density, the conventional systems^25^ demonstrate values between 12–120 Wh L^−1^, and the achieved value of 7.26 Wh L^−1^ is a promising starting point considering this as a green system.

**Fig. 16:**
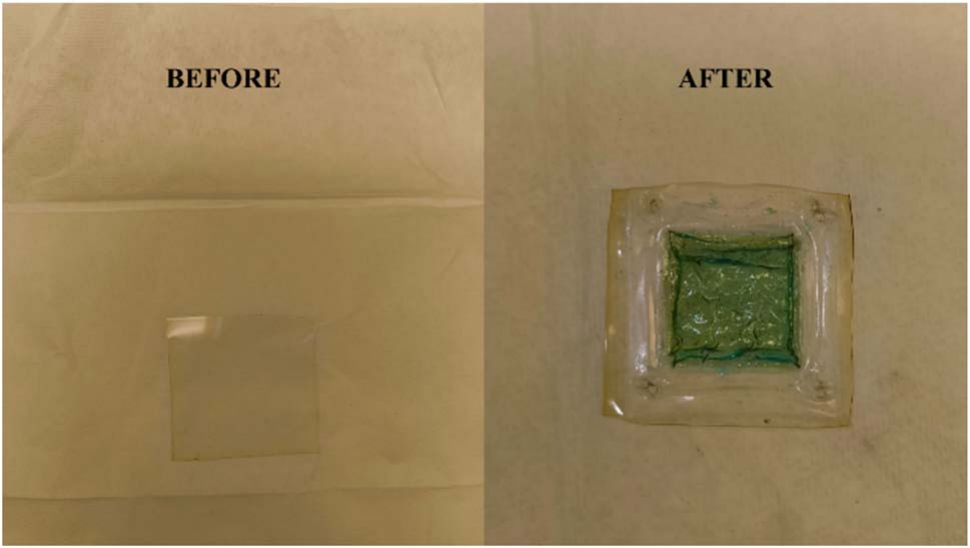
The physical condition of NAFION, a proton-exchange membrane, before and after the evaluation of charge–discharge characteristics of VRFB for numerous cycles under varying operational conditions.

Additionally, on the completion of evaluating the charge–discharge characteristics of the VRFB, the NAFION membrane has been removed from the battery to analyse its condition *i.e.*, to identify any ruptures, and the resulting images are showcased in Fig. 16. From the figure, it can be implicated that there is no damage to the membrane, and it is resistant to the synthesized formulation of aqueous ionic-liquid based electrolyte.

Furthermore, as mentioned earlier, the energy density of the VRFB can be significantly enhanced by further improving the concentration of VCl_3_ in the electrolyte while reducing the viscosity. As per the performed experiments, the maximum achievable concentration of vanadium salt utilizing BmimCl as the IL and water as solvent, was approximately 2 mol L^−1^, which can further be enhanced by the utilizing of organic solvents to reduce the viscosity and increase the concentration of vanadium salt in the electrolyte which can aid significantly in improving the performance of the battery, which will be focused as the future work.

## Conclusions

Vanadium redox flow batteries (VRFBs) hold great promise as a scalable and efficient energy storage solutions for renewable energy systems as compared to its several counterparts. However, their commercial potential has been constrained by the limited solubility of vanadium salts in the aqueous solution, leading to an increased viscosity and reduced overall operational efficiency. This study demonstrates that the incorporation of 1-Butyl-3-Methylimidazolium Chloride (BmimCl) and Vanadium Chloride (VCl_3_) in an aqueous ionic-liquid-based electrolyte can significantly enhance the solubility of the vanadium salt in DI water, resulting in improved battery performance.

The synthesized electrolyte can theoretically achieve an energy density of 44.24 Wh L^−1^, with favourable properties such as a dynamic viscosity of 36.62 mPa s at room temperature, an ionic conductivity of 0.201 S cm^−1^, and a stable potential window of approximately 1.5 V for numerous cycles. To evaluate the stability of the synthesised electrolyte, more than 200 cycles of cyclic charge–discharge has been performed. Moreover, the VRFB employing this novel electrolyte demonstrated superior cycling performance, with coulombic efficiency (CE) and capacity retention exceeding 80% at a discharge current of 5 mA. However, the formulations utilized in the VRFB, showcased poor voltage efficiency (VE) and energy efficiency (EE) owing to its ohmic, and mass-transport resistances offered by the electrolyte. These results indicate that the combination of ionic liquids and aqueous systems can overcome the solubility and viscosity limitations of conventional aqueous electrolytes, unlocking higher energy densities and greater operational stability.

The findings of this work underscore the potential of ionic-liquid based electrolytes to revolutionize VRFB technology by enhancing energy density and operational efficiency. Further exploration of organic solvents and ionic liquid combinations could lead to the development of next-generation VRFBs with higher vanadium concentrations and improved charge–discharge characteristics. This research provides a foundational step toward advanced energy storage solutions, paving the way for more efficient and sustainable grid-scale energy storage systems.

## Author contributions

Chivukula Kalyan Sundar Krishna: formal analysis, investigation, methodology, validation, visualization, writing – original draft, writing – review & editing. Yansong Zhao: conceptualization, methodology, project administration, resources, supervision, visualization, writing – review & editing.

## Conflicts of interest

There are no conflicts to declare.

## Data Availability

Data are available in the manuscript.
